# Ablation energies for focal treatment of prostate cancer

**DOI:** 10.1007/s00345-018-2364-x

**Published:** 2018-06-25

**Authors:** Olivia Lodeizen, Martijn de Bruin, Scott Eggener, Sébastien Crouzet, Sangeet Ghai, Ioannis Varkarakis, Aaron Katz, Jose Luis Dominguez-Escrig, Sascha Pahernik, Theo de Reijke, Jean de la Rosette

**Affiliations:** Olivia Lodeizen, AMC University Hospital, Meibergdreef 9, 1105 AZ Amsterdam, The Netherlands

**Keywords:** Focal therapy, Prostate cancer, Ablation, Localized

## Abstract

**Context:**

In recent years, focal therapy has emerged as a treatment option for a selected group of men with localized prostate cancer. Cryotherapy and high-intensity focused ultrasound (HIFU) are the most investigated types of focal treatment with other options currently under evaluation.

**Objective:**

The objective of the study was to give a comprehensive overview of six available focal treatment options for prostate cancer with their rationale, delivery mechanism, and outcomes.

**Information acquisition:**

The SIU ICUD chapter on available Energies to Treat Prostate Cancer was used as a guide to describe the different technologies. For outcomes, a literature search was conducted using PubMed key words including focal therapy, HIFU, cryotherapy, irreversible electroporation, vascular-targeted photodynamic therapy, laser interstitial therapy, radiofrequency ablation, microwave therapy, and their synonyms in MeSH terms.

**Conclusion:**

Focal therapy appears to have encouraging outcomes on quality of life and urinary and erectile function. For oncological outcomes, it is challenging to fully interpret the outcomes due to heterogeneity in patient selection and short-term follow-up.

**Electronic supplementary material:**

The online version of this article (10.1007/s00345-018-2364-x) contains supplementary material, which is available to authorized users.

## Introduction

Prostate cancer is increasingly diagnosed when it is still at an early, organ-confined stage due to increased awareness and improved diagnostic methods. Radiotherapy and radical prostatectomy are the most common treatments, with procedure-related morbidity ranging from 3.2 to 31% for urinary incontinence and from 58 to 79% for erectile dysfunction [1, 2]. These side effects can seriously impair quality of life. Active surveillance is appropriate for low-risk prostate cancer, but some patients have a wish for treatment and at the same time, strict compliance to active surveillance protocols appears to be difficult for patients and physicians [3, 4]. Intermediate-risk patients are typically offered active treatment, but treatment regret has been reported in ~ 15%, especially due to impaired sexual and urinary function [5, 6]. Focal therapy aims to maintain the oncological benefit of active treatment options and to reduce the risk of side effects through preserving noncancerous tissue.

Several types of energies have emerged as sources for focal ablation, investigated in a heterogeneous group of men with prostate cancer. This overview presents the available focal ablation technologies including a brief summary of their technique, procedure and (if available) functional and oncological outcomes.

For our *Outcomes* and *Recommendations,* a literature search was conducted using PubMed key words: prostate cancer, focal therapy, high-intensity focused ultrasound (HIFU), cryotherapy, irreversible electroporation (IRE), vascular-targeted photodynamic therapy (VTP), laser interstitial therapy (LITT), radiofrequency ablation (RFA), and microwave therapy. The chapter from the Société International Urologie (SIU) ICD Available Energies to Treat Prostate Cancer was used as a guide to describe the techniques. Although brachytherapy can also be used for focal therapy, due to its well-known background we will not discuss this treatment option in this article.

## High-intensity focused ultrasound ablation

### Technique

**Fig. 1:**
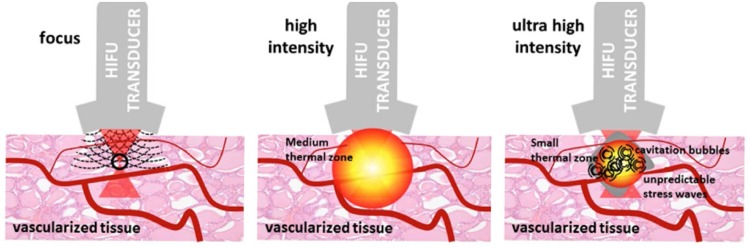
HIFU causes vascular damage at high-intensity and cavitation at ultrahigh intensity

By focusing ultrasound waves with a high-powered spherical transducer, a high-intensity beam is created, which nonspecifically ablates tissue through hyperthermia and cavitation [7]. To cause coagulative necrosis, focal temperatures between 60 and 90 °C must be achieved [8]. This process is combined with cavitation, when microbubbles will form in the tissue and implode, causing mechanical damage by disruption of the cell membrane (Fig. 1) [9]. At 3–4 MHz, HIFU lesions are visible on ultrasound as hypo-echoic areas, although ultrasound does not always accurately display their true size. MRI has improved target visualization [10].

### Device and procedure

Modern HIFU devices are used transrectally or transurethrally and are either MRI or TRUS guided. Treatment is in supine or lateral position, depending on the device. Treatment planning differs between the most commonly used devices (Supplement Table). The rectal mucosa is actively cooled, to avoid thermal damage and a catheter is placed for 1–2 weeks to avoid urinary retention due to swelling of the prostate. When combined with transurethral resection of the prostate (TURP), a catheter is placed for 2–3 days [11]. The procedure is carried out under general or spinal anesthesia in a day-care setting and takes 1–3 h, depending on prostate size. Most devices are limited to treat prostates larger than 40 cc or to lesions within 4 cm from the treatment site (rectum or urethra, respectively). HIFU is unique since it is the only true non-invasive ablative technique in prostate ablation.

### Outcome summary

Several systematic reviews assess the efficacy and safety of (whole gland and focal) HIFU in localized prostate cancer [12, 13]. A review reported by Golan et al. in 2017 included 11 studies that reported on partial HIFU in a primary treatment setting. The studies used different inclusion criteria, but included patients with a maximum < T3aN0M0 tumors with a maximum Gleason score 4 + 3. Follow-up ranged from 6 months to 10.6 years. Erectile dysfunction was reported in 0–50%. Urinary incontinence rates were reported in 0–48%. The broad range in outcomes was due to varying definitions of erectile function and continence. Eight percent of the performed follow-up biopsies were reported to have significant cancer (above Gleason 3 + 3), in either the treated or untreated lobe [12]. In 2017, a matched pair analysis of 110 men showed promising results, comparing HIFU hemiablation to robotic radical prostatectomy (RALP) in a heterogeneous group of patients with Gleason scores ≤ 6–≥ 8. HIFU was associated with a faster return to continence (no pad use) compared to RALP (82% vs 40% at 1 month, respectively), at 2 years this was comparable (94.5% HIFU vs 91% RALP). Erectile function was better in the HIFU group, with de novo persistent erectile dysfunction (measured with iPDE5) of 20% in patients treated with HIFU compared to 44% in patients treated with RALP. The need for secondary treatment was comparable in both groups, with 7 out of 55 patients in the HIFU arm and 6 out of 55 patients in the RALP arm [14].

Current IDEAL stage of research [15]: 2b

### Recommendations and ongoing trials

Overall, early evidence suggests that HIFU treatment is a safe option with varying, but mostly favorable rates of functional outcomes. Clinical trials should be performed to further investigate comorbidity of this technique, as well as the oncological control and functional outcome on a longer term. Several ongoing trials on HIFU can be found on http://www.clinicaltrials.gov, of which one investigates oncological outcomes at 6 months in 25 low- to intermediate-risk patients using hemiablation and was expected to be completed in November 2017 (NCT02016040). Results are not yet published. Another large multicenter single-arm intervention trial (the INDEX trial, *n* = 354) is expected to be completed in 2028, which will report on cancer control, erectile and urogenitary functioning (using IIEF, EPIC, IPSS and other questionnaires) and cost effectiveness (NCT01194648).

## Cryotherapy

### Technique

The rationale behind cryotherapy derives from the experiments by Thomson and Joule [16]. The sticky or repelling nature of gases can be used for temperature decrease and increase. A sticky gas in a small volume has relatively low internal energy. Once released into a larger volume through a pinhole valve, the internal energy increases due to less interaction between molecules. This energy is consumed from the environment, causing a drop in temperature. The opposite applies for repelling gases [16]. This concept is used for the freeze–thaw cycle, with fast freezing and slow thawing generating the most efficient ablation.

**Fig. 2:**
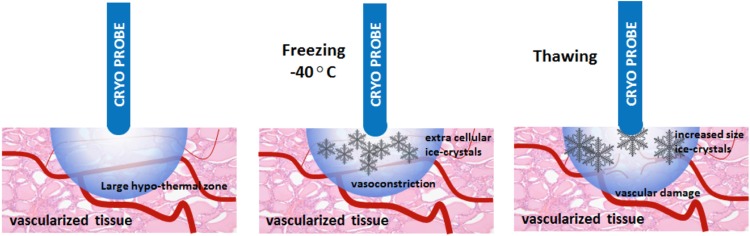
Cryotherapy causes tissue damage and vascular injury around the cryoneedle

Cryotherapy ablates all tissue in the targeted area through denaturation of cellular proteins, intracellular osmotic dehydration and metabolic failure. The resulting cellular damage is immediate, but delayed vascular injury is considered the main mechanism of cell death. Cell death occurs at − 40 °C. Initial freezing of tissue causes stasis within the blood vessels, vasoconstriction and hypoxia. Subsequent thawing restores the blood flow. However, damage to the endothelial layer continues by distension and tearing. The vascularization progressively decreases through permeability of the capillary walls, edema, platelet aggregation, and microthrombus formation [17] (Fig. 2).

### Device and procedure

**Fig. 3:**
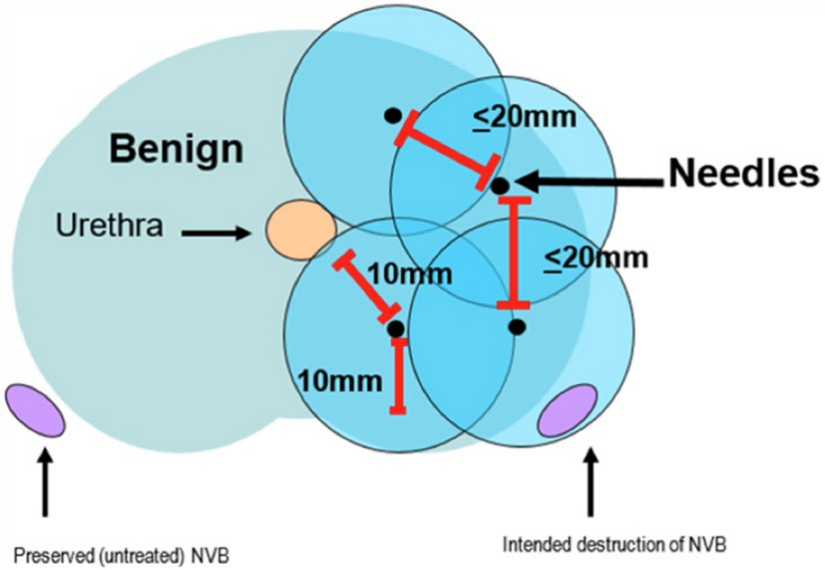
Cryoneedle placement and critical distances

**Fig. 4:**
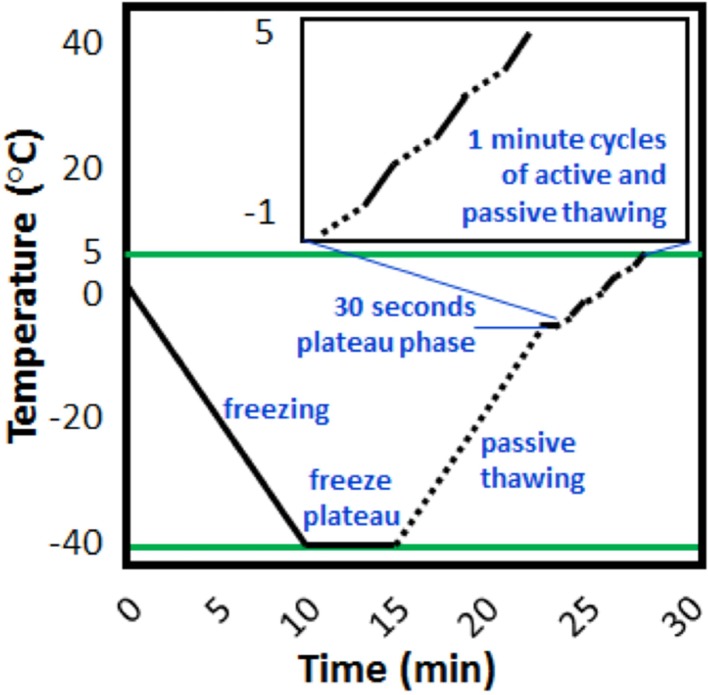
Cryotherapy stages

The cryotherapy device consists of an ultrasound-guided system, 17-gauge cryoneedles, thermocouples, and argon and helium inlets. The procedure consists of two freeze and thaw cycles. Multiple temperature probes are placed throughout the prostate and between the prostate and rectum for temperature monitoring to avoid damage. A transurethral warming device is used to prevent urethral damage. Cryoneedles are placed transperineally through a brachygrid within < 20 mm of each other (see Fig. 3), under ultrasound guidance with the patient in the lithotomy position. The needles should ideally be > 10 mm from the urethra and the posterior capsule. Typically, the ipsilateral neurovascular bundle is also ablated. The procedure can take place in an outpatient setting under spinal anesthesia. Following the procedure, a catheter is placed, which can be removed after several days. MR-guided cryotherapy has proved to allow for real-time imaging of the ablated zone by monitoring the forming of the iceball [18] (Fig. 4).

### Outcomes

In 2013, Nguyen et al. published a review comparing five studies on focal cryotherapy [19]. The studies included a varying range of patients, with low-, intermediate- and high-risk prostate cancer (PCa). The overall post-treatment incontinence was reported to be between 0 and 3.6% and potency was maintained in 58.1–90% of cases. Oncological results were not mentioned [19]. From this review, the largest study derived data from the COLD (Cryo On-Line Database) registry, with 1160 patients who underwent focal cryoablation. Control biopsies were performed when a rise in PSA occurred. Post-treatment biopsies were done in 14%, of which 26% showed in PCa. The positive biopsies were reported to have a mean Gleason score of six, which in some studies is regarded as insignificant disease. Pad-free continence was present in 98.4% and maintenance of spontaneous erections in 58.1%. Recto-urethral fistula occurred in 1 of 1160 patients [20]. Tay et al. reported on the functional and oncological results in a matched cohort analysis between partial gland and whole gland ablation in intermediate-risk prostate cancer patients (Gleason score seven or PSA > 10–20 ng/mL or clinical stage T2b). Forty eight percent of the partially ablated patients underwent biopsies, of which only 2% were positive. Indications and Gleason scores of these biopsies were not listed. After matching, 67% of men in their cohort were sufficiently potent for sexual intercourse before treatment. Among this group, 70% remained so at 12 months after partial gland ablation, compared to 45% in whole gland ablation [21].

Valerio et al. reported a systematic review on 11 studies on cryotherapy and focal therapy, of which 10 were retrospective. The patient group was heterogeneous, with inclusion criteria ranging from low- to high-risk prostate cancer, diagnosed by either transrectal or transperineal biopsies. If performed, control biopsy was done transrectally under ultrasound guidance or as targeted biopsy. The overall presence of post-treatment significant and insignificant prostate cancer was 5.4 and 13%, respectively. However, significant cancer was reported only in four series and not described homogeneously. Of all the patients in the review, 98% were leak-free continent and 100% were pad-free. Potency was maintained in 81.5%. The most common side effects were urinary retention or urinary tract infection. More serious side effects such as urethral strictures or recto-urethral fistula were rare, both reported in only 0–2.1% [22]. Current IDEAL stage of research: 2b.

### Recommendations and ongoing trials

A large amount of literature is available on cryotherapy of the prostate, primarily retrospective studies or on whole gland ablation. Partial ablation seems to have better functional outcomes than whole gland. Three ongoing prospective trials investigating the oncological and functional outcomes of focal cryotherapy in patients with (low-risk) prostate cancer are to be completed in October 2017 (NCT00774436) and 2019 (NCT00877682, NCT02459912). While cryotherapy seems to be a safe option, with reasonable functional outcomes, these prospective trials will hopefully provide more homogeneous and valuable information for its use in focal therapy of the prostate.

## Vascular-targeted photodynamic therapy

### Technique

Vascular occlusion is caused by illumination of the radical oxygen species (ROS)-generating photosensitizers with a near-infrared laser light of the tumor area. Radical oxygens (superoxide and hydroxyl) are released upon illumination, causing vascular arrest and necrosis during 24–48 h [23]. Two photosensitizers, the photosensitizer WST09 and WST11 TOOKAD^®^ Soluble, are currently available and approved. It is also known as photodynamic therapy (PDT).

### Device and procedure

**Fig. 5:**
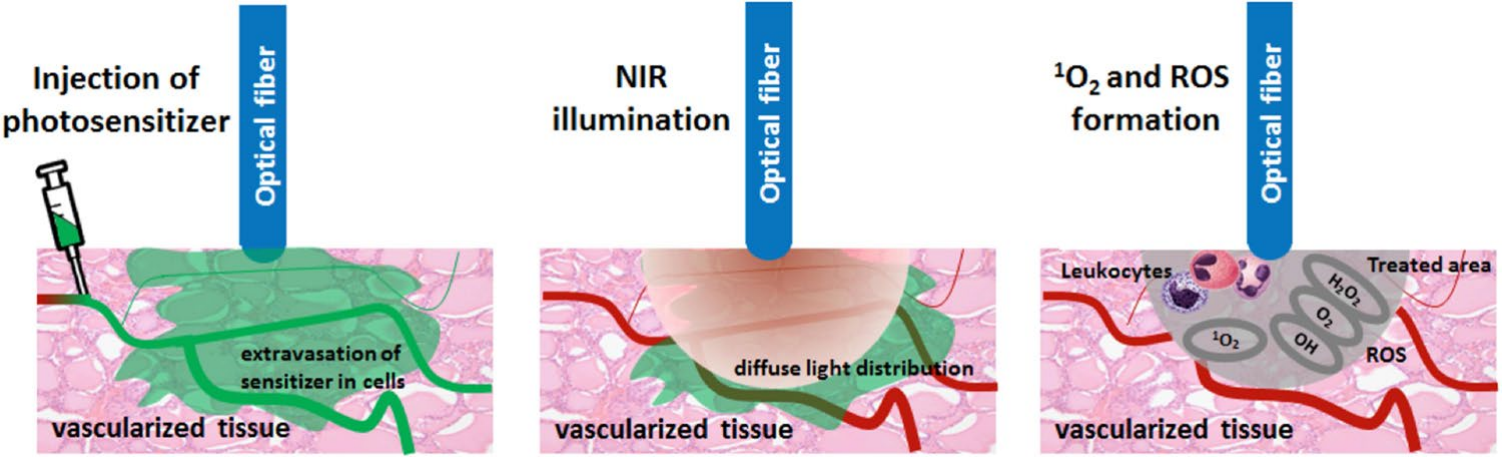
Radical oxygen causing vascular occlusion upon illumination around the fiber tips

The procedure is performed under general anesthesia in the supine position; complete muscle relaxation is advised. The fiber insertion catheters (FICs) are placed transperineally under ultrasound guidance and through a brachygrid. To avoid phototoxicity, patients must be protected from non-procedural light. A urinary catheter is placed. Once FICs are placed, with a 5 mm safety margin from the urethra, rectal wall, sphincter and capsule, the optical fibers are calibrated to adjust the delivered energy and inserted in the FICs. A single bolus of 4 mg/kg of the photosensitizer (TOOKAD^®^ Soluble) is infused intravenously for 10 min and activated by illumination with a dose light of 200 J/cm (near infrared 753 nm laser light), starting at the end of the infusion and for a period of 22 min and 15 s, matching its peak serum concentration (Fig. 5). Overall, the procedure takes about 1.5–2 h. Postoperatively, the patient is kept under dimmed light for > 6 h and then discharged after removal of the urinary catheter, avoiding direct exposure to sunlight for 48 h. An alpha-blocker can be given to diminish the risk of lower urinary tract symptoms.

### Outcome summary

Three (IDEAL Stage 1–2b) prospective development studies evaluating focal PDT in 116 patients with biopsy-proven low- and intermediate PCa have been reported in a systematic review by Valerio et al. [22]. Pre-procedural biopsies for patient selection were performed inhomogeneously. Significance of prostate cancer was not reported by these studies, but two of them reported positive biopsy rates of 24% [24] and 46% at transrectal control biopsies at 6 months [25]. Potency was maintained in 88%. One study reported 4 out of 25 patients (with baseline IIEF-5 of > 15) with de novo erectile dysfunction, defined by a decreased IIEF score of ≥ 10 points [25]. In 2017, Azzouzi et al. published results from the Phase III European RCT, including patients with low-risk (Gleason 3 + 3) PCa, comparing PDT (*n* = 206) to active surveillance (*n* = 207). With a median follow-up of 24 months, 28% had progression compared to 58% in the active surveillance (AS) group. In the PDT group, 49% had negative follow-up biopsies, compared to 14% in the AS group. Erectile dysfunction rate was 1% in both groups [26]. A more recent prospective single-arm study, by Lebdai et al., assessed oncologic outcomes of 86 men with low-risk prostate cancer. Of these patients, 77 had a Gleason score of 3 + 3, and the other five lower. During follow-up, 64 of the 82 patients (78%) underwent biopsies, either done as a routine (*n* = 20) or on clinical suspicion. Most of the biopsies were performed at 6 months (*n* = 103). The latest post-PDT data included 115 treated lobes, of which 94 (82%) did not have clinically significant prostate cancer in the treated lobes (defined as: Gleason score ≥ 7 or cancer core length greater than 3 mm regardless of grade or more than two positive cores). A Gleason score of seven or higher was found in 12 (10%) lobes, and Gleason 3 + 3 in 20 (17%). In the untreated lobes, 28 lobes (57%) were found to be positive at biopsy (Gleason score ranging from 3 + 3 to 4 + 3).

Median progression-free survival was 86 months (defined as shift into a higher-risk group according to d’Amico, which includes Gleason score greater than six, PSA 10 ng/ml and pT2b or greater). A second PDT treatment was performed in 16 of 82 men (19%). Twenty out of the 82 patients (24%) underwent radical therapy (either radical prostatectomy or brachytherapy).

Current IDEAL stage of research: 2b.

### Recommendations and ongoing trials

TOOKAD^®^ Soluble has demonstrated reasonable short- and mid-term oncological and functional outcomes. It has to be taken into account that mid-term results were reported in a low-risk group for whom the alternative would have been active surveillance, to which it was not compared. The results from Azzouzi were compared to active surveillance, but seem to have an unusually high percentage of progression in the AS group. In http://www.clinicaltrials.gov, a single-arm single-center phase IIB clinical trial is registered that will report on efficacy, safety and quality of life of 50 participants with a follow-up of 60 months. It will report on all Gleason grades of prostate cancer in treated and untreated lobes and functional outcomes using IIEF15 and IPSS. This study is expected to be completed in 2024 (NCT03315754).

## Irreversible electroporation

### Technique

Irreversible electroporation uses high-voltage low-energy electric pulses that cause cell death. These pulses travel between two or more electrodes, causing a leak in the cell membrane, formed by the creation of nanopores [27]. Depending on the field amplitude, duration and number of electrical pulses, this process can be temporary (reversible electroporation) or permanent IRE. In the case of permanent changes to the membrane, the cell will become incapable of holding on to its homeostasis and will apoptose [28].

### Device and procedure

**Fig. 6:**
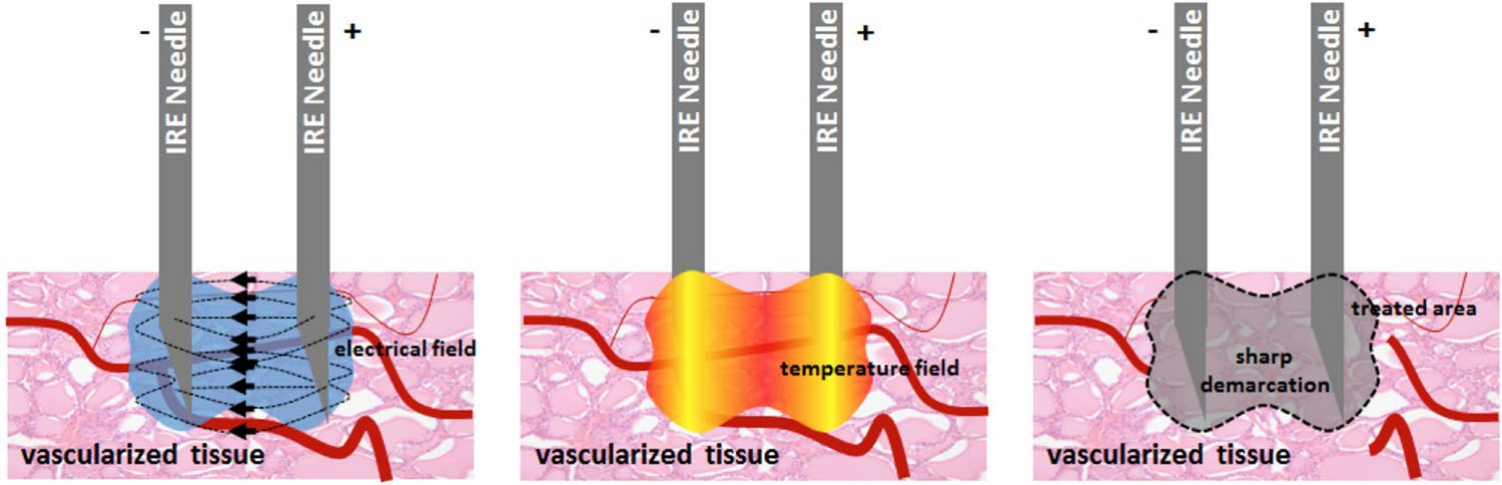
Irreversible electroporation between needles causing cell death

The IRE device consists of a low-energy direct current generator and needle-like electrode probes. Under TRUS guidance, up to six of these probes can be placed parallel at a fixed distance using a brachygrid placed to the perineum. The interprobe distance should be between 10 and 20 mm. There is one needle that activates the others, of which the size of the tip ranges from 5 to 20 mm, depending on the amount of retraction of the protective cap. This should be taken into account when deciding on the targeted ablation area. The probes should be placed at least 5 mm from the urethra, the rectum and the sphincter to avoid damage. The procedure is performed under general anesthesia in supine position, using full muscle paralysis to avoid contractions. After probe placement, appropriate parameters for voltage, number of pulses and pulse length are entered into the generator. A test pulse is first delivered to characterize the electrical current dynamics between each probe pair during ablation. Voltages are chosen to attain effective electric field strength of > 1500 Volts/cm (Fig. 6). A catheter is kept in place for > 24 h. MRI/TRUS fusion imaging techniques can be used to complement TRUS. The procedure takes approximately 45–90 min.

### Outcome summary

Ting et al. evaluated functional and oncological outcomes in 25 patients following IRE. No significant changes in urinary, sexual or bowel function were noted (using AUA scores). At follow-up there were no suspicious infield lesions on mpMRI (*n* = 24) or biopsy (*n* = 21). Adjacent to the treatment zone, five patients (21%) had suspicious lesions on mpMRI, of which four (19%) proved to be significant on biopsy. Significant prostate cancer was defined as Gleason score six with a core involvement of ≥ 5 mm and Gleason scores seven and up. Two patients (8%) had suspicious lesions on mpMRI outside the ablation zone and one (5%) a significant finding on biopsy. All patients were leak-free continent and erectile function (UCLA-EPIC) was reported to be stable [29]. Valerio et al. reported on 34 patients undergoing IRE for organ-confined prostate cancer (ranging from low- to high-risk disease). After a median follow-up of 6 months for 24 patients (range 1–24 months), 100% of patients were continent and potency was preserved in 95% (19/20) men [30]. Van den Bos et al. prospectively reported on 63 patients who received IRE treatment for organ-confined clinically significant prostate cancer (defined as high-volume Gleason score six disease and any Gleason score seven) with a minimum follow-up of 6 months. The results demonstrated no change in quality of life or mental, physical, bowel or urinary functions. A slight decrease in sexual quality of life was observed. Forty-five patients (71%) underwent control biopsies, of which 40 patients had transperineal template mapping biopsies at 6 months. Thirty-four (75%) were without significant cancer. Seven patients (16%) had infield and four patients (9%) outfield disease [31]. The reason for this high rate in positive post-procedure biopsies could be because the follow-up biopsies were performed through transperineal template mapping biopsies. A narrow safety margin of the targeted area was also reported to be a risk factor.

Current IDEAL stage of research: 2b.

### Recommendations and ongoing trials

Long-term data on oncological outcomes are still needed. The functional outcomes are promising. Ongoing trials on focal IRE in localized prostate cancer investigating functional and oncological outcomes on a longer term are awaited for future recommendations (NCT01835977).

## Laser interstitial thermotherapy

**Fig. 7:**
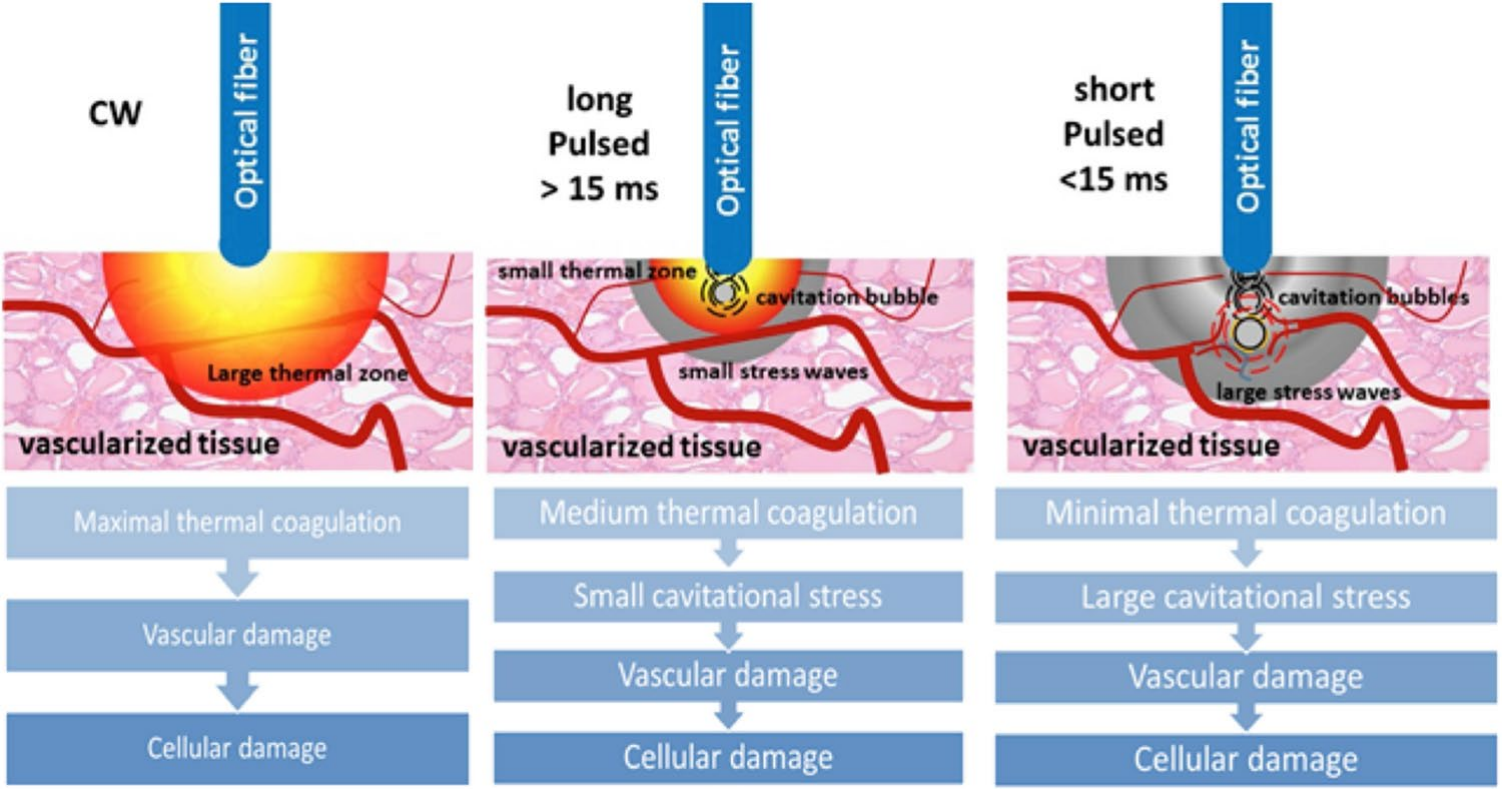
Laser therapy causing thermal damage and cavitational stress, both resulting in cellular damage

Laser interstitial thermotherapy ablates tissue through thermal damage. There are two main modes of operation: continuous wave (CW) and pulsed. A CW gives a sizeable thermal response with less control over the treatment area, whereas a pulsed laser gives a controlled thermal response with possible induction of mechanical damage by shockwaves [32] (Fig. 7).

### Device and procedure

Prior to the procedure in patients, a urinary catheter can be placed. To date, most focal laser ablation procedures have been performed under MRI guidance. The procedure can be performed in supine (transperineal) or prone position (transrectal approach). For transperineal approach, an endorectal coil helps to stabilize the prostate and a template grid is placed for needle insertion. Once the MRI-compatible titanium trocar is confirmed in position, laser applicator system consisting of a laser-diffusing fiber within a cooled catheter system is advanced to the targeted area. After reconfirming the final position of the fiber, the laser is activated to cause thermal injury. Multiplanar imaging is used to monitor critical structures such as the urethra, rectum and capsule. MR thermometry provides near real-time thermal feedback during the procedure. The duration of the procedure is dependent on the time needed to accurately target and ablate the prostate cancer lesion. Sedation with periprostatic nerve block, spinal or general anesthesia can be used. Similar to other ablative techniques, patients are discharged on the same day.

### Outcome summary

Limited clinical data are available on laser ablation, possibly due to the complicated setup and energy-consuming nature of the procedure. Lindner et al. discussed the functional and oncological results of a phase one trial, showing no significant drop in IIEF-5 or worsening of IPSS scores at 6 months post-procedure. However, at follow-up biopsies at 6 months, evidence of prostate cancer was found in 50% of patients and 67% of patients were free of tumor in the ablated zone [33]. A study of 25 men with low- and intermediate-risk prostate cancer, published by Lepor et al., demonstrated promising results regarding functional outcomes, with no significant differences between urinary (AUA scores) and erectile function (SHIM scores) at baseline and at 3 months. All men were pad-free continent. Three months following ablation, biopsies of the ablated area itself were taken and 96% showed no evidence of PCa. It is not mentioned if biopsies were taken outside the ablated area to check for treatment margins [34]. Eggener et al. reported on a prospective trial in 27 men with organ-confined PCa with Gleason ≤ 7 in ≤ 25% of pre-procedure biopsies. No significant changes in functional outcomes were observed at 12 months (IPSS and SHIM). At 12 months, ten patients had positive (transrectal ultrasound-guided 12-core) biopsies: three patients (11%) in the ablation zone and one patient in and outside the ablation zone [35].

Current IDEAL stage of research: 2b.

### Recommendations and ongoing trials

Larger series and longer-term follow-up data are needed to fully evaluate the safety and oncological outcomes. The current results are promising. Eleven ongoing trials can be found in http://www.clinicaltrials.gov that investigate focal laser therapy in prostate cancer. The Mayo Clinics started a trial in 2015 to evaluate the safety and effectiveness of MRI-guided laser therapy in 20 patients with prostate cancer tumors (T1c-cT2a and maximum Gleason score of seven). The primary outcomes are success rate of the procedure, safety by monitoring complications, incontinence, impotence and urethral fistulas (over 3 years). Secondary outcomes are short- and mid-term ablative success (over 3 years) using MRI. Estimated study completion date is December 2018. Another study initiated by the Radboud University, The Netherlands (NCT02200809), will also investigate MR-guided focal laser ablation in patients with localized intermediate-risk prostate cancer (PSA ≤ 20 ng/mL, Gleason ≤ 7, cT2b) for short- and medium-term histological cancer control, by using MR-guided biopsy results after 36 months. The estimated study completion date is July 2019.

## Radiofrequent ablation and microwave

### Technique

Radiofrequency ablation and microwave are thermal ablation techniques using radio waves. High- or medium-frequency currents cause frictional heating between ions, when dipoles realign and cause an increase in kinetic energy. Radio waves destroy tissue when temperatures rise above 50 °C for about 5 min, causing cell membrane damage, denaturation of protein and direct cytodestruction [36]. Usually, temperatures above 60 °C and higher are reached for ablation.

### Device and procedure

Prior to the procedure, patients receive a urinary catheter. RFA is performed by placing one monopolar or two bipolar needles transperineally with the patient in a supine position, under biplane transrectal ultrasound guidance. If only one needle is used, the monopolar needle is placed in the tumor. In the case of two bipolar needles, the tumor should be in-between. To monitor the temperature for ablation, a thermocouple is placed between the active bipolar needles. A thermosensor attached to the transrectal probe monitors the temperature of the rectal wall. Patients can be discharged on the same day [37].

### Outcomes

RFA and microwave therapy have mostly been studied in a salvage setting. Zlotta et al. have performed a feasibility study on RFA in 1998 in 15 patients. Of these patients, eight had an immediate radical prostatectomy following RFA treatment, and six patients underwent the procedure under spinal anesthesia followed by radical prostatectomy after 1 week. One patient had his whole prostate ablated and was followed up by PSA. The maximum temperature reached was 106 °C. The duration of the ablation itself was 10–12 min. Pathology results were not discussed, except for one patient with prostate cancer and RFA treatment in both lobes, of which tumor cells were still seen in one lobe. The urethral sphincter and rectal wall were not affected in any of the patients [37].

Current IDEAL stage of research: 2a.

### Recommendations and ongoing trials

Evidence for RFA or microwave therapy is insufficient to currently use for focal therapy. Two trials are to be completed in http://www.clinicaltrials.gov (NCT01423006 and NCT02303054), but not found on PubMed.

## Conclusion

Heterogeneity in trials has been a major limitation to comparing different ablative modalities. These limitations include, for example, heterogeneity in patient selection, method of pre- and post-procedural biopsies, questionnaires on functional outcomes and imaging. Patient selection was done using transperineal template mapping biopsies, but follow-up was performed using less (transrectal) biopsies, impeding comparison for oncological outcomes. In 2017, a panel of content experts published a consensus statement on patient selection for focal ablation of prostate cancer, advising that candidates should have localized low- to intermediate-risk disease (also including Gleason score 4 + 3). MpMRI should be used as a diagnostic tool, but systematic biopsy remains necessary to assess mpMRI-negative areas. Gleason score 3 + 3 in the untreated areas was regarded as acceptable for focal therapy [38]. Before treatment, it is advised to use template mapping biopsies to ascertain proper patient selection and to decide which areas should be ablated [39]. To make future data more comparable, it is advised to implement these consensus recommendations where possible in subsequent trials. Additional consensus should be reached for the follow-up of patients who undergo focal therapy, for example on when and how control biopsies are taken.

Within the variety of focal ablative techniques, HIFU and cryotherapy are the most thoroughly studied. HIFU has the advantage of having the most prospective trials. Regarding functional and oncological outcomes, both seem to perform equally well. Other techniques still await larger prospective trials with a longer follow-up. When compared with traditional treatment, HIFU seems to perform better in functional outcomes than RALP. It will be important, if possible, to compare focal ablation techniques to the traditional treatment options. Of all the focal techniques, most techniques seem to rely on thermal ablation. It might be a good idea to optimize one technique, rather than investigating a lot of different ones at the same time for the same purpose.

Apart from focal therapy being in an investigational stage, there are other limitations that need to be considered. An argument against focal therapy is the multifocality of prostate cancer. While there is evidence that there is an index lesion that dictates the metastatic potential [40], and therefore the targeted lesion in focal therapy, there is no definite proof of this assumption yet [41, 42]. It could be useful if studies would also report on oncological outcomes outside the treated area of the prostate.

Focal therapy in prostate cancer can be used for personalized treatment. Tumor location, number, size and wishes for functional preservation or oncological certainty should always be considered in a patient-centered manner.

The following suggestions can be given on focal therapy in prostate cancer:

1. Transurethral HIFU, LITT/laser therapy, IRE, RFA and microwave are investigational and should only be offered within clinical trials.
2. Cryotherapy and HIFU have the most data as primary treatment in organ-confined prostate cancer and can be used as primary treatment for selected patients. VTP/PDT has proved itself to be a safe option for focal treatment recently.
3. Focal ablation may be considered in a group of selected men with primary focal high-volume low- or intermediate-risk prostate cancer.
4. Identical patient selection and similar follow-up should be followed to determine the oncological and functional outcome and to allow for comparison in a high-quality systematic review on various ablation energies.

## Electronic supplementary material

Below is the link to the electronic supplementary material.

Supplementary material 1 (DOC 47 kb)
