# Supplementary material for: Ablation energies for focal treatment of prostate cancer
Source: World J Urol. 2018 Jun 25;37(3):409–18. doi: 10.1007/s00345-018-2364-x (PMC6424940; doi:10.1007/s00345-018-2364-x)
Supplement: Supplementary file 1 — Supplementary material 1 (DOC 47 kb) [file 345_2018_2364_MOESM1_ESM.doc]

| *Characteristics* | **Sonablate** | **Ablatherm** | **Focal One** | **Tulsa Pro** |
| --- | --- | --- | --- | --- |
| Set-up | Patient in supine position on operating table | Patient in lateral position on custom bed | Patient in lateral position on operating table | Patient in supine position in MRI |
| Probe | Multiple available, 25-45mm | One probe, size N/A | One probe size N/A | One transurethral |
| Frequency | 4 MHz | 3 MHz | N/A | N/A |
| Image guidance | 4 MHz | 7.5 MHz | N/A | MRI |
| Ablation planes | 3 coronal planes, anterior to posterior | Transversal 4-6 planes, apex to base | Transversal up to 8 points | Surrounding the probe |
| Ablated lesion size | focal depth away from transducer  *0.09cc - 0.11cc* | focal depth away from transducer  *0.05-0.08cc* | 5-40mm, 32-67 focal depth away from transducer | N/A |
| Whole/focal ablation | Whole gland | Focal and whole | Focal and whole | Focal and whole |
| Targeting by | US and MRI fusion imaging | US | US and MRI fusion imaging | MRI (inside gantry) |
| Feedback by | Thermometry on US | US | US, CEUS | MRI |
| Safety features | Tissue change monitoring system, color Doppler system *stacking system, reflectivity index system* | Patient motion detector, urethral cooling system, permanent control of distance to rectal wall | N/A | Thermal feedback, realtime monitoring of critical structures |
| *Protocolopties (evt)* | 1, manually adjusted | 4 (standard, re-HIFU, post-EBRT and post-brachytherapy) | N/A | N/A |
| Manufacturer | SonaCare Medical,  Indianapolis, United States | EDAP TMS S.A., Vaulx-en-Velin, France | EDAP TMS S.A., Vaulx-en-Velin, France | Profound Medical, Mississauga, |

| ***Device and manufacturer*** | **Visual Ice, Galil Medical inc**® | **Cryocare, Endocare Inc®** |
| --- | --- | --- |
| Set-up | Supine position, needles trough brachygrid and perineum | Supine position, needles trough brachygrid and perineum |
| Image guidance | Ultrasound or CT | Ultrasound |
| Needles | 1,5-2,4mm tip length | Up to 6 needles, adjustable in length |
| Safety | Thermal sensors | Thermal sensors |

| ***Ablation type*** | **IRE** | **Laser** | **Radiofrequency ablation** |
| --- | --- | --- | --- |
| ***Device and manufacturer*** | NanoKnife® IRE System, AngioDynamics | Visualase®, Medtronic | RITA medical systems, In 2007 bought by AngioDynamics (now StarBurst®) |
| Set-up | Supine position under general anesthesia and rocuronium, needles trough brachygrid and perineum | In MRI gantry, single catheter through perineum | Supine position under general anesthesia, needle through perineum |
| Image guidance | Ultrasound | MRI | Ultrasound  (MRI and CT reported) |
| Needles | 2-6 needles, 0.5-2.0 cm tiplength | 1.65mm flexible catheter with 360° diffusing laser tip | 8-16mm diameter bipolar probe |
| Safety | Cardiac monitor | In-plane thermometry, temperature limit possible to set | N/A |
